# Broad-Based CD4^+^ T Cell Responses to Influenza A Virus in a Healthy Individual Who Lacks Typical Immunodominance Hierarchy

**DOI:** 10.3389/fimmu.2017.00375

**Published:** 2017-04-03

**Authors:** Li Chen, Anjaleena Anthony, Sara Oveissi, Miaojuan Huang, Damien Zanker, Kun Xiao, Chao Wu, Quanming Zou, Weisan Chen

**Affiliations:** ^1^National Engineering Research Center of Immunological Products, Department of Microbiology and Biochemical Pharmacy, College of Pharmacy, Third Military Medical University, Chongqing, China; ^2^T Cell Laboratory, School of Molecular Science, La Trobe Institute of Molecular Science, La Trobe University, Bundoora, VIC, Australia; ^3^Department of Blood Transfusion, The Second Affiliated Hospital, The Third Military Medical University, Chongqing, China

**Keywords:** influenza A virus, CD4^+^ T cell, immunodominance, epitope, HLA

## Abstract

Influenza A virus (IAV) infection is a significant cause of morbidity and mortality worldwide. CD4^+^ T cell responses have been shown to be important for influenza protection in mouse models and in human volunteers. IAV antigen-specific CD4^+^ T cell responses were found to focus on matrix 1 (M1) and nucleoprotein (NP) at the protein antigen level. At the epitope level, only several epitopes within M1 and NP were recognized by CD4^+^ T cells. And the epitope-specific CD4^+^ T cell responses showed a typical immunodominance hierarchy in most of the healthy individuals studied. In this study, we reported one case of atypical immunodominance hierarchy of CD4^+^ T cell responses to IAV. M1 and NP were still the immunodominant targets of CD4^+^ T cell responses. However, CD4^+^ T cell responses specific to 11 epitopes derived from M1 and NP were detected and showed no significant immunodominance hierarchy. Such an atypical pattern is likely determined by the individual’s HLA alleles. These findings will help us better understand the anti-IAV immunity as a whole and improve future vaccines against IAV.

## Introduction

Influenza virus [influenza A virus (IAV)] infection is a global threat to human health. Each year, about half a billion human beings have symptomatic influenza illness (1), and three to five million subjects suffer from severe influenza, causing approximately half a million deaths annually worldwide (2). Frequent mutation in hemagglutinin and neuraminidase of the circulating viruses and the mismatch between the circulating and vaccine viruses significantly affected the effectiveness of antibody-based vaccine strategy (3). Novel vaccines that are more effective and covering a broader spectrum of influenza viruses are urgently needed. T cell immunity has an important protective role against IAV, and T cell-based vaccines represent an important new development, worldwide, in efforts to combat influenza (4).

Study of IAV-specific T cell immunity has focused more on CD8^+^ T cells (5, 6) partly due to the lack of accurate prediction algorithms for CD4^+^ T cell epitopes that often show promiscuous length requirement (7). Otherwise, specific CD4^+^ T cell responses were proven to be indispensable for the clearance of IAV in both animal models (8, 9) and in human volunteers (10). CD4^+^ T cells can exert their protective effect directly through cytotoxic activity (11) or indirectly through providing “help” to both CD8^+^ T cells and B-cells to eliminate virus and virus-infected cells *via* cytotoxicity and antibody neutralization, respectively (12, 13). Furthermore, the generation of strong memory CD4^+^ and CD8^+^ T cell responses are also CD4^+^ helper T cell dependent (14, 15). Thus, stimulating robust CD4^+^ T cell response is critical for both developing effective T cell-based and antibody-based IAV vaccine (16). To realize that and to be able to properly appreciate the future IAV vaccine efficiency, antigen specificity of IAV-specific CD4^+^ T cell responses need to be properly understood and finely characterized.

Immunodominance refers to the phenomenon that the cellular immunity tends to focus on a very limited number of antigenic epitopes even during immune responses to complex antigens or pathogens in infected individuals. Immunodominance in CD4^+^ T cell responses have been widely observed in many viral systems, including HIV, EBV, HTLV1, and others (17–19) and such immunodominance hierarchies are often long lasting (20). Using *in vitro* expanded multi-specificity IAV-specific T cell lines and individual IAV protein antigens produced by recombinant vaccinia viruses (rVVs), we have demonstrated that matrix 1 (M1) and nucleoprotein (NP) are the immunodominant antigens targeted by IAV-specific CD4^+^ T cells in healthy individuals (21). We further finely characterized 10 immunodominant epitopes derived from these antigens using synthetic overlapping peptides (21). Although some of these have been previously reported, their immunodominance status was confirmed for the first time. The epitope-specific CD4^+^ T cell responses showed a typical immunodominance hierarchy in most of the healthy individuals we studied. In some individuals, the CD4^+^ T cell responses even focused on a single epitope (21).

In the present study, using the same approach as mentioned above, we found atypical CD4^+^ T cell responses to IAV in a healthy individual. Although M1 and NP were still the immunodominant targets of these CD4^+^ T cell responses and up to 11 epitopes derived from nine antigenic regions were recognized, the magnitude of these epitope-specific CD4^+^ T cell responses were relatively equal, and no significant immunodominance was observed. From this, one highly conserved epitope, M1_240–252_ restricted to DPB1*0501, was identified. The potential implication of these findings to T cell-based vaccine development is further discussed.

## Materials and Methods

### PBMC Samples

Buffy coats were obtained with informed written consent from the Australian Red Cross donors under the agreement of 12-07VIC-17 Material Supply Agreement V15.1. PBMC were isolated by Ficoll-Hypaque gradient and stored in liquid nitrogen until use. HLA typing was performed by the Victorian Transplantation and Immunogenetics Service (VTIS, Melbourne, VIC, Australia). The proposed work was approved by the Faculty of Science, Technology & Engineering Human Ethics Committee under the project number FHEC12/NR81.

### Synthetic Peptides

All peptides were synthesized by Mimotopes (Melbourne, VIC, Australia); IAV-M1 and NP overlapping 18mers with 6-aa shifts, and 13mers with either 1- or 2-aa shifts were synthesized as cleaved peptide libraries. All peptides were dissolved in DMSO.

### Viruses

The Mount Sinai strain of PR8 (A/Puerto Rico/8/1934 H1N1) IAV was prepared as previously described (21). Virus aliquots were stored at −80°C until use. rVV for the generation of individual IAV proteins were gifts from Drs. Jonathan Yewdell and Jack Bennink (National Institutes of Health, Bethesda, MD, USA). The viruses were propagated using a TK^−^ cell line and were stored at −80°C until use. These proteins are all derived from the PR8 sequences.

### Cell Culture

Donor EBV BLCLs (Epstein–Barr virus-transformed B lymphoblast cell lines) were established using standard EBV transformation. The other human BLCL lines were made available from the International HLA Workshop and the Victorian Transplantation and Immunogenetics Service (Melbourne, VIC, Australia). P815 cells were kind gifts from Drs. Jonathan Yewdell and Jack Bennink (National Institutes of Health, Bethesda, MD, USA). All cells were cultured in RF-10 consisting of RPMI-1640 supplemented with 10% FCS, 2-ME (5 × 10^−5^ M), and antibiotics (penicillin 100 U/mL, streptomycin 100 µg/mL).

### Preparation of IAV- and rVV-Infected P815 Cell Lysates

Influenza A virus and rVV infection of P815 cells were conducted as previously described (21). Infected cells were pelleted and lysed by 8 M urea. The lysates were aliquoted and preserved at −20°C until use.

### Generating IAV-Specific, Polyspecificity CD4^+^ T Cell Lines

PBMCs (5 × 10^6^) were pulsed with 5 µL IAV-infected P815 cell lysates (equivalent to 10^5^ infected cells) in 200 µL RF-10 for 1 h in 24-well tissue culture plates. Two microliters RF-10 with 20 U/mL recombinant human interleukin-2 (rIL-2) (Peprotech, Brisbane, QLD, Australia) were then added, and the cell lines were cultured in the rIL-2-containing RF-10 until use.

### Generating Single Peptide-Specific CD4^+^ T Cell Lines

Peptide-specific CD4^+^ T cell lines were generated as previous described (21, 22). In brief, PBMCs (1–2 × 10^6^) were pulsed with 5 µM peptide and cultured in 1 mL “RP-5” consisting of RPMI 1640 (Gibco) supplemented with 5% human AB sera, l-glutamine (2 mM), 2-ME (5 × 10^−5^ M), and antibiotics in 48-well tissue culture plates. The medium was 50% replaced by RP-5 containing 10 U/mL rIL-2 on day 5 and then 50% replaced by RP-5 containing 20 U/mL rIL-2 when required.

### Identification of Antigenic Regions and Epitopes

IFN-γ intracellular cytokine staining (ICS) was performed to identify antigenic regions and epitopes as previously described (21). In brief, autologous BLCLs were pulsed with IAV- or rVV-infected P815 cell lysates overnight and then cocultured with IAV-specific T cell lines for 5 h in the presence of 10 µg/mL Brefeldin A (BFA) or single peptide-specific T cell cultures were incubated with peptide at 10 µg/mL at 37°C for 5 h in the presence of BFA. First, the cells were harvested and stained with anti-CD3 (FITC) and anti-CD4 (APC) and then washed, fixed, and stained with anti-IFN-γ (PE-Cy7) as described previously (23). The flow cytometry mAbs were purchased from eBioscience. Samples were acquired on a FACS Canto II flow cytometer (Becton Dickinson), and FACS data were analyzed with FlowJo software (Tree Star, Ashland, OR, USA).

### HLA Restriction Assay

For antibody-blocking assay, T cells were incubated with 10 µL of anti-HLA class II antibody for 30 min before addition of peptide and BFA. Pan anti-DR (L243), anti-DP (B7/21), and anti-DQ (SPV-L3) antibodies were used as culture supernatants (22). For identifying restriction HLA, BLCLs were pulsed with the peptide of interest at 10 µg/mL for 1 h, washed extensively, and then cocultured with peptide-specific T cells for 5 h in the presence of BFA. Then, IFN-γ ICS was performed as described above.

### Bioinformatics Analysis

Protein sequences were aligned and amino acid differences were scored to determine the sequence conservation between IAV strains for the newly identified peptides. The National Center for Biotechnology Information (NCBI) Influenza virus database^1^ was used (accessed on November 7, 2016) with the search criteria set as Australia, M1/NP, H1N1/H3N2 [or Any (Country/region), M1/NP, H5N1] identical sequences were represented by the oldest sequence in the group and full length only, which identified H1N1 (*n* = 19 for M1, *N* = 43 for NP), H3N2 (*n* = 24 for M1 and *n* = 74 for NP), and H5N1 (*n* = 36 for M1 and *n* = 96 for NP) sequences. Protein sequences were aligned using the NCBI database, peptide regions were mapped, and frequency of mutation was determined across the various sequence groups.

## Results

### Approach for Systematical Identification of IAV-Specific CD4^+^ T Cell Responses

**Figure 1:**
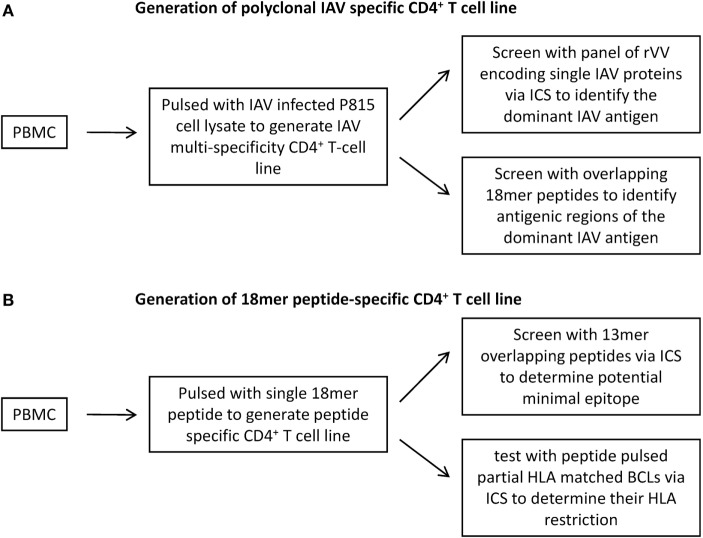
**Generation of CD4^+^ T cell lines for the identification of antigen specificity of Influenza A virus (IAV)-specific CD4^+^ T cell responses**. **(A)** To identify the dominant IAV antigen, multi-specificity IAV-specific CD4^+^ T cell lines were generated by pulsing PBMCs with IAV-infected P815 cell lysates and then cultured in the presence of rIL-2 for 12–15 days. The immunodominant IAV antigens were identified using an IFN-γ intracellular cytokine staining (ICS) assay in response to autologous BLCLs pulsed with recombinant vaccinia viruses (rVV)-infected P815 cell lysates, which were engineered to express a single IAV protein. Following the identification of immunodominant antigens, antigenic regions were determined by 18mer overlapping peptides covering the corresponding antigens. **(B)** To identify the epitopes buried in the antigenic regions, single 18mer peptide-specific CD4^+^ T cell lines were generated and screened for 13mer overlapping peptides using ICS assay. The HLA restriction was identified by partially HLA-matched BLCLs pulsed with target peptide in an ICS assay.

A two-step approach (21) is established for systematic identification of IAV-specific CD4^+^ T cell responses. Step one is the identification of the dominant IAV antigen that stimulated CD4^+^ T cell responses (Figure 1A), and then, step two is the determination of potential minimal sequence of epitopes together with their HLA restriction (Figure 1B). To identify the dominant virus protein, multi-specificity, IAV-specific CD4^+^ T cell lines were generated by stimulating PBMCs in the presence of IL-2 with a soluble IAV antigen source generated by lysing IAV-infected P815 cells in 8 M urea. The cell lines were then screened with a panel of lysates generated by P815 cells infected with rVV, which were engineered to express individual IAV proteins (21, 23, 24). To further identify the immunodominant epitope regions within the dominant proteins, 18mer overlapping peptides covering the full protein sequence were screened by using an ICS assay measuring interferon-γ production (Figure 1A). Next, the same PBMCs were stimulated with 18mer peptides covering the immunogenic regions to establish single epitope-specific T cell lines. The potential minimal epitope sequences were determined using ICS assays in response to overlapping 13mer peptides. The HLA restrictions were identified by HLA class II-blocking antibodies and further confirmed by partially HLA-matched antigen-presenting cell (APC) lines (Figure 1B).

### M1 and NP Are Dominant Antigens Recognized by IAV-Specific CD4^+^ T Cells

**Figure 2:**
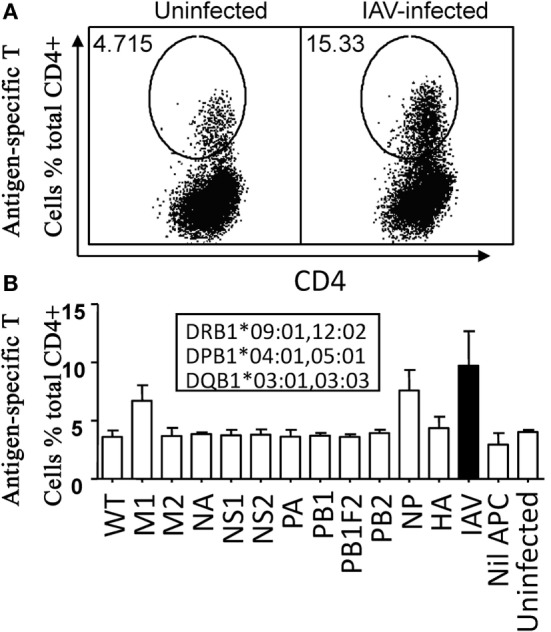
**M1 and nucleoprotein (NP) were dominant antigens recognized by Influenza A virus (IAV)-specific CD4^+^ T cells**. The IAV-specific T cell line was generated by IAV-infected P815 cell lysate. Approximately, 12–15 days later, the cells were tested for their reactivity to autologous BLCLs pulsed with individual lysate of P815 cells infected with IAV or the 11 recombinant vaccinia viruses (rVVs) encoding single IAV antigen in an IFN-γ intracellular cytokine staining assay. BLCLs not pulsed with any lysate (Nil), or pulsed with lysate from uninfected P815 cells (Uninfected), wild type (WT, empty vector) rVV-infected P815 cells were used as background and specificity controls. Representative dot plots were shown in panel **(A)**. Histogram of all individual responses was shown in panel **(B)**. Total IAV-specific CD4^+^ T cell response stimulated by autologous BLCL pulsed with IAV-infected P815 cell lysate was shown in black bars for easier comparison. Donor’s HLA-class II alleles were shown in the inset text boxes. The test was repeated for three times of independent T cell cultures by two researchers. The error bars indicate the standard error of the mean.

To identify dominant antigens recognized by IAV-specific CD4^+^ T cells, multi-specificity IAV-specific CD4^+^ T cell lines were generated by stimulating PBMCs with a urea dissolved soluble IAV antigen (Figure 2A). In response to 12 rVV (11 rVVs encoding 11 individual IAV proteins including PB1-F2 and one wild type rVV)-infected P815 lysates, only M1 and NP stimulated specific IFN-γ producing CD4^+^ T cells over background (Figure 2B). Therefore, M1 and NP were dominant targets recognized by IAV-specific CD4^+^ T cells in this donor.

### Atypical Immunodominance Hierarchy of IAV-Specific CD4^+^ T Cell Responses

**Figure 3:**
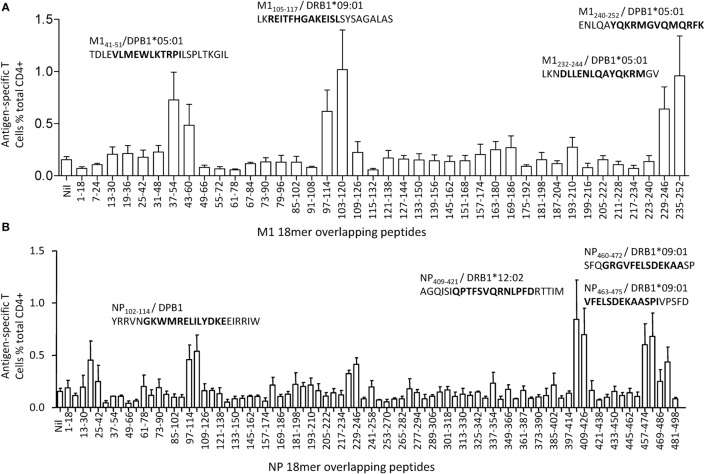
**Identification of antigenic regions within M1 and nucleoprotein (NP)**. The same influenza A virus-specific T cell lines used in Figure 2 were further screened for their specific response to the 121 overlapping 18mer peptides from M1(40) and NP(81) at a final concentration around 1 µg/mL in an intracellular cytokine staining assay. The identified 18mer sequences are shown, and the subsequently identified epitopes and their HLA restrictions are bolded. Antigenic regions derived from M1 were shown in panel **(A)**, while antigenic regions derived from NP were shown in panel **(B)**. The screening was repeated for three times of independent T cell cultures by two researchers. The error bars indicate the SEM.

M1 and NP have been demonstrated to be the most dominant targets of IAV-specific CD4^+^ T cell responses in healthy individuals by others (25) and by us (21), and it seemed no exception in this donor. To further define IAV-specific CD4^+^ T cell responses in this donor, M1 and NP 18mer overlapping peptides were screened using the multi-specificity T cell line. As shown in Figure 3, unlike most of the IAV-specific CD4^+^ T cell responses generally focusing on one or two antigenic regions and displaying a typical immunodominance hierarchy (21), no significant immunodominant region was observed in this donor although up to nine antigenic regions in M1 and NP were recognized. The magnitudes of the CD4^+^ T cell responses revealed by the 18mer peptides were relatively equal, including M1(37–60), M1(97–120), M1(229–252), NP19–42, NP97–120, NP223–246, NP403–426, NP457–480, and NP469–492. Thus, there was no typical immunodominance hierarchy of IAV-specific CD4^+^ T cell responses observed in this subject.

### Fine Characterization of Epitopes Derived from M1 and NP

As MHC is one of the important determining factors of the immunodominance, to further explore such an atypical immunodominance hierarchy of IAV-specific CD4^+^ T cell responses, potential minimal sequences of epitopes were determined by overlapping 13mer peptides within the antigenic 18mer regions. To further increase the accuracy of epitope identification, the antigenic neighboring 13mers with single amino acid difference in sequence were quantitatively assessed by the T cell lines in a peptide titration assay. HLA restriction was further identified.

**Figure 4:**
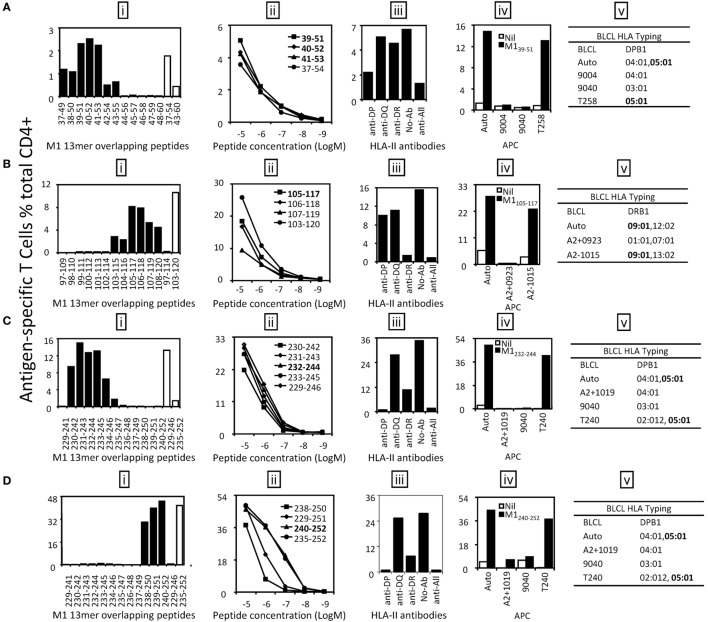
**Identification of the core sequences and HLA restrictions of the epitopes derived from M1**. 18mer peptide-specific T cell lines were established to identify the core sequences and HLA restrictions of the epitopes. **(A)** The 13mer peptides within 18mer M1(37–54) and M1(43–60) were screened by intracellular cytokine staining (ICS) (i) and the control 18mer results are shown by open bars. Several potential key 13mers and corresponding 18mers were titrated in FCS-containing condition to compare their T cell stimulating capacity, which led to the identification of the core peptide M1_39–51_ (ii). HLA restriction of M1_39–51_ was then determined by HLA-class II antibody blocking assay (iii) and partial HLA matching BLCLs (iv, v). **(B)** The 13mer peptides within 18mer M1(97–114) and M1(103–120) were screened by ICS (i). M1_105–117_ was titrated to be the core peptide (ii). HLA restriction of M1_105–117_ was determined (iii–v). **(C)** The 13mer peptides within 18mer M1(229–246) were screened (i). The core 13mer peptide M1_232–244_ was identified by titration (ii), and HLA restriction of M1_232–244_ was analyzed (iii–v). **(D)** The 13mer peptides within 18mer M1(235–252) were screened as in panel **(A)** (i). The core 13mer peptide M1_240–252_ was identified by titration (ii), and its HLA restriction was determined (iii–v). Some of the assays were performed after the T cell lines were restimulated *in vitro* for two to three times.

Three antigenic regions were identified from M1 protein (Figure 3A). The CD4^+^ T cells responding to the M1(37–54) and M1(43–60) 18mer peptides (Figure 3A) recognized seven 13mer peptides [Figure 4A (i)]. Among them, M1(39–51), M1(40–52), and M1(41–53) stimulated similar responses and the titration of these three 13mers showed almost identical potency. We, therefore, consider M1_41–51_ is the core and minimal epitope sequence as it is shared by all three peptides and as M1(38–50) and M1(42–54) were much less potent [Figure 4A (ii)]. To determine the restricting HLA molecule for M1_41–51_, a class II antibody-blocking assay was first conducted. The anti-DP antibody efficiently blocked most T cell activation to peptide M1(39–51), whereas the anti-DR and anti-DQ antibodies did not [Figure 4A (iii)]. To further confirm the HLA-DP restriction of M1_41–51_, a panel of BLCLs with different DP alleles [Figure 4A (iv, v)] was used as APCs after being pulsed with M1(39–51) to stimulate the peptide-specific T cell line. Autologous BLCL and BLCL T258 both expressing HLA-DPB1*05:01 efficiently activated peptide-specific T cells. In contrast, BLCL 9004 and 9040 do not express HLA-DPB1*05:01 and failed to present this peptide [Figure 4A (iv, v)]. Therefore, the M1_41–51_-specific CD4^+^ T cell response is restricted to HLA-DPB1*05:01. Using the same approach, M1_105–117_ restricted to HLA-DRB1*09:01 (Figure 4B) was identified from antigenic region M1(97–120). While within the antigenic region M1(229–252), there were two different epitopes identified: M1_232–244_ (Figure 4C) and M1_240–252_ (Figure 4D). Although they were both restricted to HLA-DPB1*05:01 (Figures 4C,D), no cross-reactivity was observed [Figure 4C (i); Figure 4D (i)].

**Figure 5:**
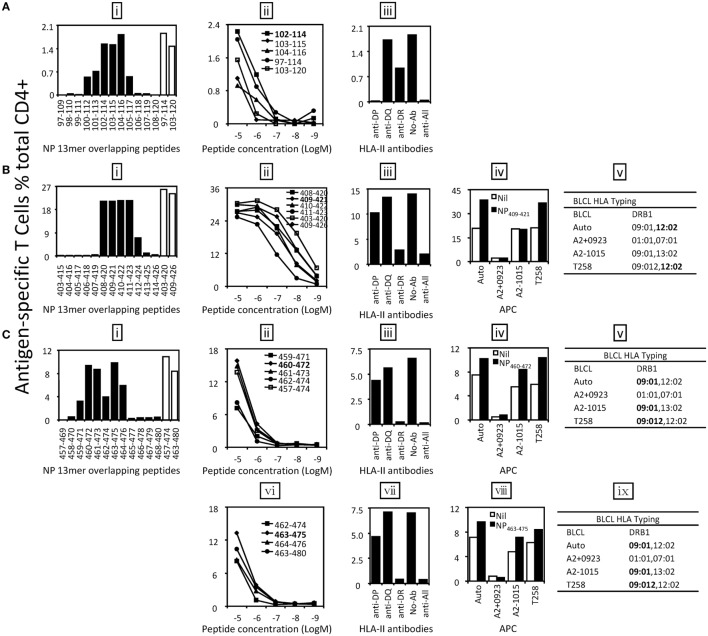
**Identification of the core sequences and HLA restrictions of epitopes derived from nucleoprotein (NP)**. 18mer peptide-specific T cell lines were established to identify the core sequences and HLA restrictions of epitopes. **(A)** The 13mer peptides within 18mer NP97–114 and NP103–120 were screened by intracellular cytokine staining (ICS) (i) and the control 18mer results are shown by open bars. Several potential core 13mers and two 18mers were titrated to compare their T cell stimulating capacity, which led to the identification of the core peptide NP_102–114_ (ii). HLA restriction of NP_102–114_ was then determined by HLA-class II antibodies (iii). **(B)** The 13mer peptides within 18mer NP403–420 and NP409–426 were screened by ICS (i) and the corresponding 18mer results are shown by open bars. Several 13mers and two 18mers were titrated (ii). HLA restriction of the core 13mer NP_409–421_ was determined by HLA-class II antibodies (iii) and partial HLA matching BLCLs (iv, v). **(C)** The 13mer peptides within 18mer NP457–474 and NP463–480 were screened and 18mer results are shown by open bars (i). Four overlapping 13mer peptides and NP457–474 were titrated to compare their activity (ii), and HLA restriction of the core 13mer NP_460–472_ was analyzed with HLA-class II antibodies (iii) and partial HLA-matched BLCLs (iv, v). Another core 13mer peptide NP_463–475_ was identified by titration (vi), and its HLA restriction was determined (vii–ix). Some of the assays above were performed after the T cell lines were restimulated by the same 18mer or core 13mer for two to three times.

Six antigenic regions were identified in NP protein (Figure 3B). Among them, four epitopes within three antigen regions were finely characterized. The epitope in antigenic region NP97–120 was shown to be NP_102–114_ [Figure 5A (i, ii)]. It was shown to be restricted to HLA-DP [Figure 5A (iii)]; however, we were not able to further resolve whether that was HLA-DPB04:01- or 05:01-restricted. Epitope NP_409–421_ restricted to HLA-DRB1*12:02 was identified within the antigenic region NP403–426 (Figure 5B). Two independent epitopes, NP_460–472_ and NP_463–475_, were identified in the antigenic region NP457–480, and interestingly, both restricted to HLA-DRB1*09:01 (Figure 5C). The epitopes in the remaining three antigenic regions, NP19–42, NP223–246, and NP469–492, were not finely characterized due to limited PBMC availability.

## Discussion

In this study, we report one case of broad IAV-specific CD4^+^ T cell response in one healthy individual using a systematic approach (Figure 1). We demonstrate that M1 and NP are still the immunodominant targets of CD4^+^ T cell responses (Figure 2). The 18mer-screen identified nine antigenic regions containing at least 11 epitopes, among which eight have been finely characterized for their core sequences and HLA restriction (Table 1). A few previously reported epitopes were among the ones we identified in this study, such as M1_105–117_, NP_102–114_, and NP_463–475_. However, some of these such as M1_33–52_, M1_228–244_, NP_102–114_, and NP_409–421_ were reported to be restricted to different HLA-II molecules; for example NP_409–421_ peptide restricted to DRB1*0801 rather than here DRB1*1202 (26). Moreover, M1_240–252_ restricted to DPB1*0501, was identified as a highly conserved epitope among strains of H1N1, H3N2, and even H5N1 (Table 2).

**Table 1:** **Epitopes identified in this study and previously reported epitopes containing the same sequences**.

Identified epitopes	Identified epitope sequences	HLA restriction	Reported epitopes	Reported epitope sequences	Reference	HLA restriction	Method
M1_41–51_	VLMEWLKTRPI	DPB1*05:01	M1_33–52_	AGKNTDLEVLMEWLK TRPIL	(27)	DRB1*12:01	Multimer/tetramer qualitative binding
M1_105–117_	REITFHGAKEISL	DRB1*09:01	M1_105–117_	REITFHGAKEISL	(21)	DRB1*09:01	Intracellular cytokine staining (ICS) IFNg release
			M1_105–117_	REITFHGAKEISL	IEDB	DRB1*07:01	Multimer/tetramer qualitative binding
			M1_105–124_	REITFHGAKEISLSYSAGAL	(28)	DRB1*01:03	ELISPOT IFNg release
M1_232–244_	DLLENLQAYQKRM	DPB1**05:01	M1_228–244_	GLKNDLLENLQAYQKRM	(29)	DRB5	ELISPOT IFNg release
M1_240–252_	YQKRMGVQMQRFK	DPB1*05:01	M1_240–252_	YQKRMGVQMQRFK	(29)	DQ1	ELISPOT IFNg release
NP_102–114_	GKWMRELILYDKE	DP	NP_102–114_	GKWMRELILYDKE	(21)	DPB1*01:01	ICS IFNg release
NP_409–421_	QPTFSVQRNLPFD	DRB1*12:02	NP_409–426_	QPTFSVQRNLPFDKTTIM	(10)	HLA-class II	ELISPOT IFNg release
			NP_409–428_	QPTFSVQRNLPFDRT TIMAA	IEDB	DRB1*15:01	Multimer/tetramer qualitative binding
			NP_409–428_	QPTFSVQRNLPFDRT TIMAA	(26)	DRB1*08:01	Multimer/tetramer qualitative binding
NP_460–472_	GRGVFELSDEKAA	DRB1*09:01	Not reported previously				
NP_463–475_	VFELSDEKAASPI	DRB1*09:01	NP_463–475_	VFELSDEKAASPI	(21)	DRB1*09:01	ICS IFNg release

**Table 2:** **Conservancy of the peptide sequences within H1N1, H3N2, and H5N1 viruses**.

Peptide	Peptide sequence	Frequency (%) of peptide variants		
		H1N1	H3N2	H5N1
M1_41–51_	VLMEWLKTRPI^a^	–	4	–
	A··········	100	92	100
	A········S·	–	4	–
M1_105–117_	REITFHGAKEISL^a^	–	4	–
	···········A·	32	96	–
	··········V··	63	–	6
	····L······A·	5	–	–
	··········VA·	–	–	64
	··M·······VA·	–	–	25
	··K·······VA·	–	–	3
	····Y·····VA·	–	–	3
M1_232–244_	DLLENLQAYQKRM^a^	84	83	–
	···D·········	11	–	–
	···K·········	5	–	–
	N············	–	4	64
	N··········T·	–	–	6
	N··D·········	–	–	6
	·······A·····	–	13	–
	··I··········	–	–	25
M1_240–252_	YQKRMGVQMQRFK^a^	95	100	80
	········I····	5	–	–
	····H········	–	–	14
	···X·········	–	–	3
	············R	–	–	3
NP_102–114_	GKWMRELILYDKE^a^	67	3	–
	·······V·····	2	61	–
	···V···V·····	30	–	2
	·R·····V·····	–	30	–
	·······V····K	–	1	–
	·······V····D	–	1	–
	·······V····G	–	1	–
	·R·V···V·····	–	1	–
	·····G·V·····	–	1	–
	···V·········	–	–	98
NP_409–421_	QPTFSVQRNLPFD^a^	40	3	–
	··A··········	–	8	–
	············E	56	89	96
	·········S···	4	–	1
	··N·······F··	–	–	1
	····L····S···	–	–	2
NP_460–472_	GRGVFELSDEKAA^a^	5	8	–
	············T	60	92	91
	··········R·T	33	–	–
	··········R··	2	–	–
	······F·····T	–	–	8
	········V···T	–	–	1
NP_463–475_	VFELSDEKAASPI^a^	5	1	–
	·········TN··	60	88	91
	·········TN·V	–	4	–
	·······R··N··	2	–	–
	·······R·TN··	33	–	–
	···F·····TN··	–	–	8
	·····V···TN··	–	–	1
	··········N··	–	7	–

*Australian circulated H1N1 [*n* = 19 for M1, *N* = 43 for nucleoprotein (NP)] and H3N2 (*n* = 24 for M1 and *n* = 74 for NP) sequences included the full-length sequences of viruses available from the National Center for Biotechnology Information (NCBI) influenza virus resource database accessed on November 7, 2016. Search criteria were Australia, M1/NP, H1N1/H3N2, identical sequences were represented by the oldest sequence in the group, full length only*.

*H5N1 without country/region restriction (*n* = 36 for M1 and *n* = 96 for NP) sequences included the full-length sequences of viruses available from the NCBI influenza virus resource database accessed on November 7, 2016. Search criteria were any (country/region), M1/NP, H5N1, identical sequences were represented by the oldest sequence in the group, full length only*.

*^a^These peptide sequences were identified and used in this study*.

The term immunodominance, although originally defined as a restricted T cell response to a peptide from a given protein (30), is generally applicable to CD8^+^ and CD4^+^ T cell response to many infections. The mechanisms associated with it, although intensely studied for decades, are still not fully understood both at cellular level and at organism level. In our previous study, a typical immunodominance hierarchy was observed in IAV-specific CD4^+^ T cell responses to the dominant antigens M1 and NP; and within the dominant antigen, only one or two epitopes were selected to stimulate dominant CD4^+^ T cell responses (21). However, the IAV-specific CD4^+^ T cell responses in this study, although also focused on M1 and NP (Figure 2B), showed clearly an atypical pattern as all the detected 11 epitopes derived from nine antigenic regions of M1- and NP-stimulated CD4^+^ T cell responses to a comparable level (Figure 3).

Multiple determinants, especially peptide generation, DM-mediated peptide exchange (editing), and responding T cell repertoire are involved in the establishment of immunodominance hierarchy (31). However, the most important determining element, often under discussed, is restricting MHC as in the syngeneic murine systems, the MHC is pre-fixed and often seeming outside the consideration. In humans, it is widely reported that the T cell responses differ between individuals with different HLA alleles. That is also well demonstrated in IAV-specific CD8^+^ (23, 24) and CD4^+^ T cell responses (21). NP_463–475_ and M1_105–117_, previously identified in one healthy individual [donor 1 in Ref. (21)], was confirmed here as restricted to DRB1*09:01 (Table 3). However, M1_94–106_ restricted to DRB1*13:02 and was not selected to stimulate a CD4^+^ T cell response in this donor as DRB1*13:02 was not expressed (Table 3). Instead, NP_409–421_ restricted to DRB1*12:02 was detected (Figure 5B). Further, three DPB1*05:01-restricted epitopes, M1_39–51_, M1_232–244_, and M1_240–252_ were identified in this donor. Interestingly, although T cells specific to NP_463–475_/DRB1*09:01 was dominant in our previous donor 1 (21), it is on par with other responses detected in the current donor (Figure 3). These results indicate that all DRB1*09:01, DPB1*05:01, and DRB1*12:02 are HLA alleles that play almost equal role in shaping the anti-IAV CD4^+^ T cell response in this individual resulting in broad CD4^+^ T cell response pattern without pronounced immunodominance.

**Table 3:** **Comparison of antigenic regions and epitopes of Influenza A virus-specific CD4^+^ T cells between the donor in this study and the donor 1 in our previous publication (21)**.

Donor	HLA-II alleles	Antigenic regions	Epitopes	HLA restriction	Immunodominance status
Donor in this study	DRB1*09:01,12:02	M1(37–60)	M1_39–51_	DPB1*05:01	N/A
	DPB1*04:01,05:01	M1(97–120)	M1_105–117_	DRB1*09:01	N/A
	DQB1*03:01,03:03	M1(229–252)	M1_232–244_	DPB1*05:01	N/A
			M1_240–252_	DPB1*05:01	N/A
		NP(19–42)	NT	NT	N/A
		NP(97–120)	NP_102–114_	DP	N/A
		NP(223–246)	NT	NT	N/A
		NP(403–426)	NP_409–421_	DRB1*12:02	N/A
		NP(457–480)	NP_460–472_	DRB1*09:01	N/A
			NP_463–475_	DRB1*09:01	N/A
		NP(469–492)	NT	NT	N/A

Donor 1 previously reported in Ref. (21)	DRB1*09:01,13:02	M1(91–108)	M1_94–106_	DRB1*13:02	SDD
	DPB1*01:01,04:01	M1(97–120)	M1_105–117_	DRB1*09:01	SDD
	DQB1*03:03,06:04	NP(1–24)	NT	NT	SDD
		NP(97–120)	NP_102–114_	DPB1*01:01	SDD
		NP(223–246)	NT	NT	SDD
		NP(403–426)	NT	NT	SDD
		NP(457–480)	NP_463–475_	DRB1*09:01	IDD

Donor 4 previously reported in Ref. (21)	DRB1*01:01,07:01	M1(127–144)	M1_129–141_	DRB1*01:01	IDD
	DPB1*04:01,05:01				
	DQB1*02:01,05:01				

*SDD, subdominant determinant; IDD, immunodominant determinant*.

It is possible that the infection history or even previous vaccination may influence the T cell response, and therefore the immunodominance hierarchy in the studied samples. However, without knowing the exact IAV exposure history of the individual, it would be difficult to approach this concern using just a few IAV strains as stimulating antigens. To our knowledge, there is no report showing a systematical evaluation of one individual’s IAV T cell response using various IAV strains.

We believe that the HLA combination in an individual is very important in determining the outcome of CD4^+^ T cell immunodominance hierarchy. For example, when DPB1*05:01 was co-expressed with DRB1*01:01, the dominant response to IAV changed to M1_129–141_/DRB1*01:01 [donor 4 in Ref. (21)]. It seems that DRB1*01:01 has the priority to present dominant epitopes when DRB1*01:01 was co-expressed with other HLA-class II alleles. This was partially supported by the data in the Immune Epitope Database (IEDB).^2^ So far, 88 IAV epitopes restricted to DRB1*01:01 were indexed, while only six epitopes were reported to be restricted to DRB1*09:01, and no epitope was found to be DPB1*05:01 restricted, indicating that DRB1*01:01 might play a bigger role in presenting IAV epitopes than many other HLA alleles.

Many IAV-derived CD4^+^ T cell epitopes have been identified and indexed in the IEDB.^3^ As shown in Table 1, some epitopes identified in the present study were reported previously. However, we found a portion of the epitopes restricted to more than one HLA allele. For example, M1_232–244_ and M1_240–252_, although reported to be restricted to DRB5 and DQ1, respectively (29), were found in our study to be presented by DPB1*0501 (Figures 4C,D); NP_409–421_ restricted to DRB1*12:02 (Figure 5B) was once reported to be restricted to DRB1*15:01 (IEDB) and DRB1*08:01 (26), etc. These results further confirmed that many CD4^+^ T cell epitopes may be presented by multiple HLA molecules (Table 1). However, since most of the previously identified epitopes were not defined to their minimal core sequences, the reported sequences might contain two or more different epitopes restricted to various HLA molecules. All of the epitopes, including the novel epitope NP_460–472_, identified in the present study were defined to their most potent core sequences by 13mer overlapping peptides and peptide titration. Interestingly, we also identified an epitope M1_240–252_/DPB1*05:01, previously reported to be restricted to DQ1, located in the C-terminal of M1. It is highly conserved in H1N1, H3N2, and even H5N1 strains (Table 2), indicating that these 13 amino acids are likely critical for M1 binding to ribonucleocapsids. DPB1*05:01 was highly expressed in the population of Australia Cape York Peninsula Aborigine (45.3%) and Kimberly Aborigine (68.4%) based on the data of HLA Allele Frequencies Database.^4^ The Aboriginal population has been shown to be more susceptible to influenza (32). Thus, this might be a good candidate for IAV vaccine development in the Australian Aborigine population.

In conclusion, using a systematic screening approach, we confirmed that IAV-specific CD4^+^ T cell responses in the studied individual focus on M1 and NP as we previously reported (21). Eight epitopes were finely characterized for their core sequences, HLA restriction, and sequence conservation. Interestingly, the broad T cell responses were largely equal, and we failed to observe the typical immunodominance hierarchy. We believe HLA allele expression might be the major mechanism that leading to such broad-based and less-focused CD4^+^ T cells response as in principle immunodominance hierarchy (and potentially the lack of it) should be HLA-dependent. To the best of our knowledge, no such atypical pattern of immunodominance hierarchy was reported in CD4^+^ T cell responses to IAV before. The identification of such immune response pattern may help us further understand cellular immunity against IAV and development of T cell-based vaccines.

## Author Note

In the text, all published and defined minimal epitopes are shown as subscribed amino-acid positions, such as NP_463–475_ or M1_39–51_; other peptide sequences are shown as normal text, such as M1(37–60) or NP19–42.

## Author Contributions

LC designed and performed experiments, analyzed the data, and wrote the manuscript. AA, SO, and MH performed experiments and analyzed the data. DZ performed experiments, analyzed the data, and wrote the manuscript. KX performed experiments. CW designed experiments and wrote the manuscript. QZ designed experiments and wrote the manuscript.

## Conflict of Interest Statement

The authors declare that the research was conducted in the absence of any commercial or financial relationships that could be construed as a potential conflict of interest.

## Footnotes

^1^http://www.ncbi.nlm.nih.gov/genomes/FLU/Database/nph-select.cgi?go=1.

^2^http://www.iedb.org/home_v3.php.

^3^www.iedb.com.

^4^http://www.allelefrequencies.net/hla6006a.asp.

## References

[1] Hayward AC Fragaszy EB Bermingham A Wang L Copas A Edmunds WJ Comparative community burden and severity of seasonal and pandemic influenza: results of the Flu Watch cohort study. Lancet Respir Med (2014) 2(6):445–54. 10.1016/S2213-2600(14)70034-7 24717637 PMC7164821

[2] Vaccines against influenza WHO position paper – November 2012. Wkly Epidemiol Rec (2012) 87(47):461–76. 23210147

[3] Puig-Barbera J Burtseva E Yu H Cowling BJ Badur S Kyncl J Influenza epidemiology and influenza vaccine effectiveness during the 2014-2015 season: annual report from the global influenza hospital surveillance network. BMC Public Health (2016) 16(Suppl 1):757 10.1186/s12889-016-3378-1 27556802 PMC5001209

[4] Grant EJ Chen L Quinones-Parra S Pang K Kedzierska K Chen WS. T-cell immunity to influenza A viruses. Crit Rev Immunol (2014) 34(1):15–39. 10.1615/CritRevImmunol.2013010019 24579700

[5] Hamada H Bassity E Flies A Strutt TM Garcia-Hernandez Mde L McKinstry KK Multiple redundant effector mechanisms of CD8+ T cells protect against influenza infection. J Immunol (2013) 190(1):296–306. 10.4049/jimmunol.1200571 23197262 PMC3864858

[6] Sun J Braciale TJ. Role of T cell immunity in recovery from influenza virus infection. Curr Opin Virol (2013) 3(4):425–9. 10.1016/j.coviro.2013.05.001 23721865 PMC3804899

[7] Chaves FA Lee AH Nayak JL Richards KA Sant AJ The utility and limitations of current web-available algorithms to predict peptides recognized by CD4 T cells in response to pathogen infection. J Immunol (2012) 188(9):4235–48. 10.4049/jimmunol.1103640 22467652 PMC3331894

[8] Belz GT Wodarz D Diaz G Nowak MA Doherty PC. Compromised influenza virus-specific CD8(+)-T-cell memory in CD4(+)-T-cell-deficient mice. J Virol (2002) 76(23):12388–93. 10.1128/JVI.76.23.12388-12393.2002 12414983 PMC136883

[9] Teijaro JR Njau MN Verhoeven D Chandran S Nadler SG Hasday J Costimulation modulation uncouples protection from immunopathology in memory T cell responses to influenza virus. J Immunol (2009) 182(11):6834–43. 10.4049/jimmunol.0803860 19454679

[10] Wilkinson TM Li CK Chui CS Huang AK Perkins M Liebner JC Preexisting influenza-specific CD4+ T cells correlate with disease protection against influenza challenge in humans. Nat Med (2012) 18(2):274–80. 10.1038/nm.2612 22286307

[11] Babon JA Cruz J Ennis FA Yin L Terajima M. A human CD4+ T cell epitope in the influenza hemagglutinin is cross-reactive to influenza A virus subtypes and to influenza B virus. J Virol (2012) 86(17):9233–43. 10.1128/jvi.06325-11 22718815 PMC3416118

[12] DiPiazza A Richards KA Knowlden ZA Nayak JL Sant AJ. The role of CD4 T cell memory in generating protective immunity to novel and potentially pandemic strains of influenza. Front Immunol (2016) 7:10. 10.3389/fimmu.2016.00010 26834750 PMC4725218

[13] Brown DM Lampe AT Workman AM. The differentiation and protective function of cytolytic CD4 T cells in influenza infection. Front Immunol (2016) 7:93. 10.3389/fimmu.2016.00093 27014272 PMC4783394

[14] Sun JC Bevan MJ. Defective CD8 T cell memory following acute infection without CD4 T cell help. Science (2003) 300(5617):339–42. 10.1126/science.1083317 12690202 PMC2778341

[15] Shedlock DJ Shen H. Requirement for CD4 T cell help in generating functional CD8 T cell memory. Science (2003) 300(5617):337–9. 10.1126/science.1082305 12690201

[16] Devarajan P Bautista B Vong AM McKinstry KK Strutt TM Swain SL. New insights into the generation of CD4 memory may shape future vaccine strategies for influenza. Front Immunol (2016) 7:136. 10.3389/fimmu.2016.00136 27148257 PMC4827017

[17] Goon PK Igakura T Hanon E Mosley AJ Barfield A Barnard AL Human T cell lymphotropic virus type I (HTLV-I)-specific CD4+ T cells: immunodominance hierarchy and preferential infection with HTLV-I. J Immunol (2004) 172(3):1735–43. 10.4049/jimmunol.172.3.1735 14734756

[18] Stenger RM Poelen MC Moret EE Kuipers B Bruijns SC Hoogerhout P Immunodominance in mouse and human CD4+ T-cell responses specific for the *Bordetella pertussis* virulence factor P.69 pertactin. Infect Immun (2009) 77(2):896–903. 10.1128/IAI.00769-08 19015250 PMC2632029

[19] Ning RJ Xu XQ Chan KH Chiang AK. Long-term carriers generate Epstein-Barr virus (EBV)-specific CD4(+) and CD8(+) polyfunctional T-cell responses which show immunodominance hierarchies of EBV proteins. Immunology (2011) 134(2):161–71. 10.1111/j.1365-2567.2011.03476.x 21896011 PMC3194224

[20] Jing LC Schiffer JT Chong TM Bruckner JJ Davies DH Felgner PL CD4 T-cell memory responses to viral infections of humans show pronounced immunodominance independent of duration or viral persistence. J Virol (2013) 87(5):2617–27. 10.1128/Jvi.03047-12 23255792 PMC3571405

[21] Chen L Zanker D Xiao K Wu C Zou Q Chen W. Immunodominant CD4+ T-cell responses to influenza A virus in healthy individuals focus on matrix 1 and nucleoprotein. J Virol (2014) 88(20):11760–73. 10.1128/JVI.01631-14 25078703 PMC4178733

[22] Chen L Li B Yang WC He JL Li NY Hu J A dominant CD4+ T-cell response to helicobacter pylori reduces risk for gastric disease in humans. Gastroenterology (2013) 144(3):591–600. 10.1053/j.gastro.2012.12.002 23232294

[23] Wu C Zanker D Valkenburg S Tan B Kedzierska K Zou QM Systematic identification of immunodominant CD8+ T-cell responses to influenza A virus in HLA-A2 individuals. Proc Natl Acad Sci U S A (2011) 108(22):9178–83. 10.1073/pnas.1105624108 21562214 PMC3107317

[24] Grant E Wu C Chan KF Eckle S Bharadwaj M Zou QM Nucleoprotein of influenza A virus is a major target of immunodominant CD8(+) T-cell responses. Immunol Cell Biol (2013) 91(2):184–94. 10.1038/icb.2012.78 23399741

[25] Lee LY Ha do LA Simmons C de Jong MD Chau NV Schumacher R Memory T cells established by seasonal human influenza A infection cross-react with avian influenza A (H5N1) in healthy individuals. J Clin Invest (2008) 118(10):3478–90. 10.1172/JCI32460 18802496 PMC2542885

[26] Chow IT James EA Gates TJ Tan V Moustakas AK Papadopoulos GK Differential binding of pyruvate dehydrogenase complex-E2 epitopes by DRB1*08:01 and DRB1*11:01 is predicted by their structural motifs and correlates with disease risk. J Immunol (2013) 190(9):4516–24. 10.4049/jimmunol.1202445 23543758 PMC3729472

[27] Chow IT James EA Tan V Moustakas AK Papadopoulos GK Kwok WW. DRB1*12:01 presents a unique subset of epitopes by preferring aromatics in pocket 9. Mol Immunol (2012) 50(1–2):26–34. 10.1016/j.molimm.2011.11.014 22196942 PMC3433834

[28] Yang J James EA Huston L Danke NA Liu AW Kwok WW. Multiplex mapping of CD4 T cell epitopes using class II tetramers. Clin Immunol (2006) 120(1):21–32. 10.1016/j.clim.2006.03.008 16677863

[29] Babon JA Cruz J Orphin L Pazoles P Co MD Ennis FA Genome-wide screening of human T-cell epitopes in influenza A virus reveals a broad spectrum of CD4(+) T-cell responses to internal proteins, hemagglutinins, and neuraminidases. Hum Immunol (2009) 70(9):711–21. 10.1016/j.humimm.2009.06.004 19524006 PMC2767101

[30] Sercarz EE Lehmann PV Ametani A Benichou G Miller A Moudgil K Dominance and crypticity of T cell antigenic determinants. Annu Rev Immunol (1993) 11:729–66. 10.1146/annurev.iy.11.040193.003501 7682817

[31] Kim A Sadegh-Nasseri S Determinants of immunodominance for CD4 T cells. Curr Opin Immunol (2015) 34:9–15. 10.1016/j.coi.2014.12.005 25576665 PMC4444386

[32] Miller A Durrheim DN Aboriginal and Torres Strait Islander Community Influenza Study Group Aboriginal and Torres Strait Islander communities forgotten in new Australian National Action Plan for human influenza pandemic: “ask us, listen to us, share with us”. Med J Aust (2010) 193(6):316–7. 20854233 10.5694/j.1326-5377.2010.tb03939.x

